# Recent advances in various adeno-associated viruses (AAVs) as gene therapy agents in hepatocellular carcinoma

**DOI:** 10.1186/s12985-024-02286-1

**Published:** 2024-01-12

**Authors:** Meead Hadi, Omer Qutaiba B. Allela, Mansoureh Jabari, Asna Mahyazadeh Jasoor, Omid Naderloo, Saman Yasamineh, Omid Gholizadeh, Leila Kalantari

**Affiliations:** 1 Department of Microbiology, Faculty of Basic Science, Central Tehran Branch, Islamic Azad University, Tehran, Iran; 2 Department of Pharmacy, Al-Noor University College, Nineveh, Iraq; 3 Medical Campus, Xi’an Jiaotong University, Xi’an, Shaanxi Province China; 4 Department of Microbiology, School of Biology, College of Science, University of Tehran, Tehran, Iran; 5 Department of Laboratory Sciences, Faculty of Medicine, Islamic Azad University of Gorgan Breanch, Gorgan, Iran; 6 Virology & Biotechnology, Tehran, Iran; 7 School of Medicine, Kashan University of Medical Sciences, Kashan, Iran

**Keywords:** Adeno-associated virus, Hepatocellular carcinoma, Gene therapy, Hepatitis

## Abstract

Primary liver cancer, which is scientifically referred to as hepatocellular carcinoma (HCC), is a significant concern in the field of global health. It has been demonstrated that conventional chemotherapy, chemo-hormonal therapy, and conformal radiotherapy are ineffective against HCC. New therapeutic approaches are thus urgently required. Identifying single or multiple mutations in genes associated with invasion, metastasis, apoptosis, and growth regulation has resulted in a more comprehensive comprehension of the molecular genetic underpinnings of malignant transformation, tumor advancement, and host interaction. This enhanced comprehension has notably propelled the development of novel therapeutic agents. Therefore, gene therapy (GT) holds great promise for addressing the urgent need for innovative treatments in HCC. However, the complexity of HCC demands precise and effective therapeutic approaches. The adeno-associated virus (AAV) distinctive life cycle and ability to persistently infect dividing and nondividing cells have rendered it an alluring vector. Another appealing characteristic of the wild-type virus is its evident absence of pathogenicity. As a result, AAV, a vector that lacks an envelope and can be modified to transport DNA to specific cells, has garnered considerable interest in the scientific community, particularly in experimental therapeutic strategies that are still in the clinical stage. AAV vectors emerge as promising tools for HCC therapy due to their non-immunogenic nature, efficient cell entry, and prolonged gene expression. While AAV-mediated GT demonstrates promise across diverse diseases, the current absence of ongoing clinical trials targeting HCC underscores untapped potential in this context. Furthermore, gene transfer through hepatic AAV vectors is frequently facilitated by GT research, which has been propelled by several congenital anomalies affecting the liver. Notwithstanding the enthusiasm associated with this notion, recent discoveries that expose the integration of the AAV vector genome at double-strand breaks give rise to apprehensions regarding their enduring safety and effectiveness. This review explores the potential of AAV vectors as versatile tools for targeted GT in HCC. In summation, we encapsulate the multifaceted exploration of AAV vectors in HCC GT, underlining their transformative potential within the landscape of oncology and human health.

## Introduction

Liver cancer, encompassing hepatocellular carcinoma (HCC), is recognized as the sixth most often occurring cancer and is identified as the third leading cause of cancer-related deaths worldwide [1]. Surgical resection, trans-arterial chemoembolization, liver transplantation, and radiofrequency ablation are effective tactics for managing early-stage HCC. However, HCC often receives its diagnosis when it has progressed, leaving limited choices for addressing advanced-stage cases [2–4]. Furthermore, their non-specific impact on cancer cells has a broad spectrum of adverse effects. Within several months, patients undergoing these treatments develop resistance, necessitating the shift to second-line therapies like regorafenib, pembrolizumab, nivolumab, and cabometyx. Given these drawbacks, the implementation of gene therapy (GT) for HCC emerges as an appealing alternative [5].

Patients grappling with HCC face an urgent demand for novel therapeutic approaches, and GT emerges as a captivating avenue, holding substantial potential primarily attributed to the distinctive circulatory arrangement of the liver. Notably, the liver predominantly relies on the portal vein for the bulk of its blood supply, with a mere 25% originating from the hepatic artery. In stark contrast, HCC tumors procure over 95% of their blood supply from the hepatic artery [6, 7]. Consequently, the administration of gene therapeutics faces a unique advantage within this context. Ordinarily constrained by challenges in attaining tumor-localized concentrations, gene therapies achieve a heightened degree of specificity for HCC through direct intra-arterial injection into the liver. Moreover, the integration of this approach with trans-arterial chemoembolization, a well-established technique in HCC treatment, holds the potential to amplify the extent of therapeutic payload transportation to the tumor site [8]. While the liver stands as an optimal target due to its distinct vascular characteristics, diverse delivery vectors have been investigated to surmount the challenges inherent in gene delivery [9]. Numerous alternative viral vectors have exhibited promise for GT in HCC. Nevertheless, the Adeno-associated virus (AAV) is a well-established and widely acknowledged gene delivery vector for liver-related conditions [9]. AAV vectors offer significant benefits due to their infrequent genome integration and minimal genotoxicity [10]. AAV2, 5, 8, 9, and 3B are utilized in gene therapies targeted at the human liver based on their natural tissue preference (tropism), whereas AAV2, 9, rh10, and rh8 are administered locally or intravenously for therapies involving the central nervous system (CNS). Currently, preclinical applications of AAV vectors designed for liver and CNS GT are also being observed in the context of malignancies originating from the liver or CNS [11]. AAV vectors of nearly all serotypes exhibit effective liver accumulation upon intravenous administration, owing to their inherent affinity for the liver (hepatic tropism) [12]. AAV serotype 3 is a potent choice for effectively delivering genetic material to human liver cancer cells. This is due to AAV3’s utilization of the human hepatocyte growth factor receptor as a co-receptor for attaching to and entering these cells. Consequently, AAV3 vectors hold promise for application in liver cancer GT. Furthermore, while AAV8-based vectors demonstrate a notable 10- to 100-fold higher efficiency in transducing mouse livers compared to AAV2- or AAV5-based vectors, substantial preclinical and clinical evidence suggests that AAV8-based vectors, widely employed in current clinical practices, do not effectively and selectively target primary human hepatocytes. Additionally, findings from preclinical models lack predictability regarding their performance in human clinical scenarios [13, 14]. Additionally, an AAV6 serotype, tailored for cancer immunotherapy based on dendritic cells (DCs), presents a promising strategy for effectively targeting and treating cancer models [15]. Notably, almost all natural AAV capsids demonstrate an adequate capacity to transduce hepatic tissue after systemic administration. As a result, recombinant adeno-associated viruses (rAAVs) present a robust platform geared towards precise liver targeting, affording opportunities for addressing an array of ailments, including HCC, hemophilia A, and B, familial hypercholesterolemia, Crigler–Najjar syndrome, and ornithine transcarbamylase deficiency [16].

Here, we will delve into the function of AAV vectors and provide an extensive overview of the latest advancements and the potential applications of this vector system within the rapidly progressing field of cancer research.

## Characteristics of different AAVs as gene therapy vectors

**Fig. 1:**
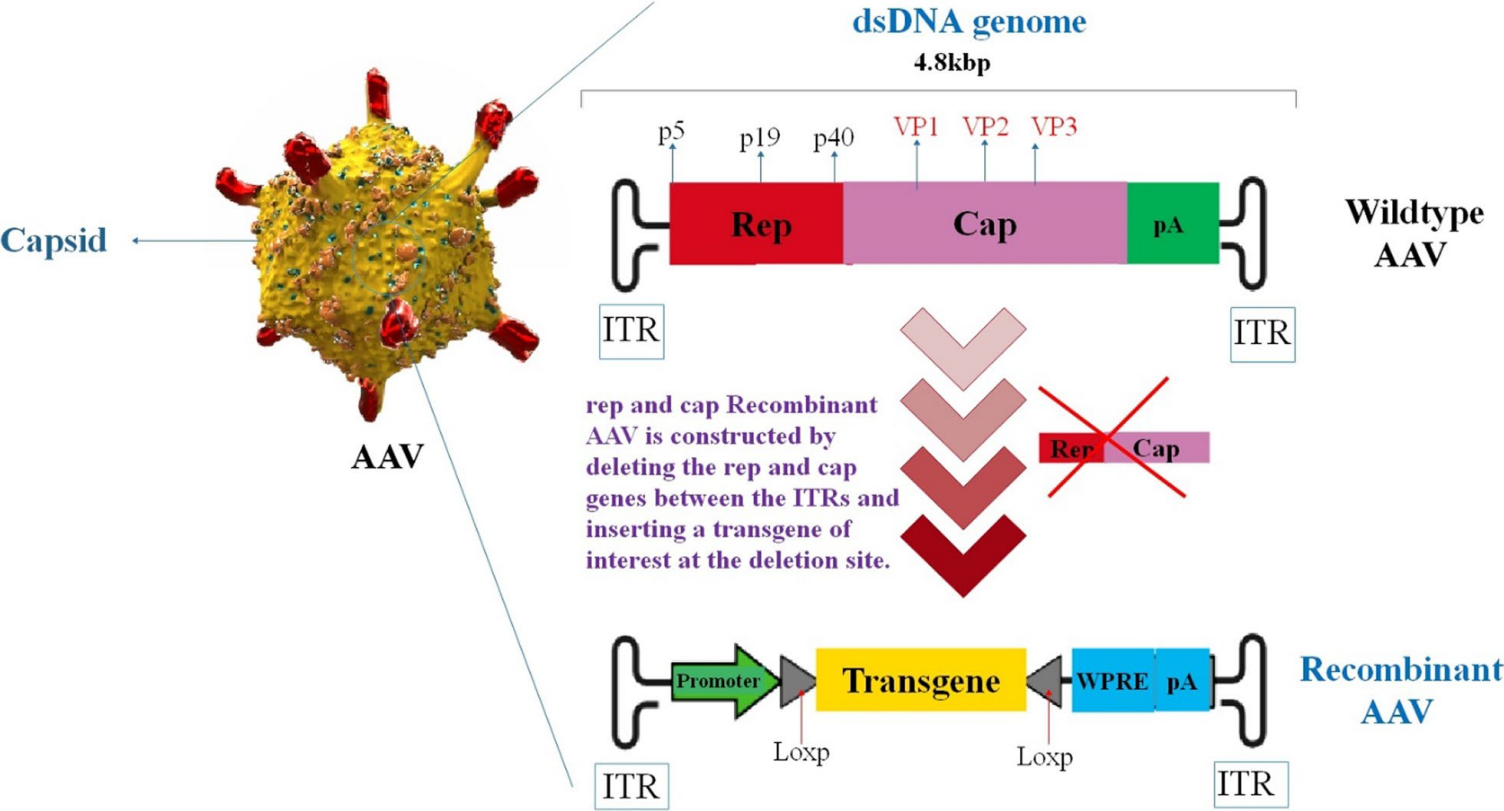
A Genome structure of wild-type AAV and recombinant AAV. Wild-type AAV genomes consist of inverted terminal repeats (ITRs) that flank two open reading frames (ORFs), rep and cap. To generate recombinant AAV, the rep and cap genes are deleted between the ITRs, followed by the insertion of a transgene of interest at the deletion site. In trans, the rep and cap functions are implemented when the viral vector is manufactured. Rep58 and Rep68 are encoded by the p5 promoter, whereas Rep52 and Rep40 are encoded by the p19 promoter as part of the structure of the wild-type AAV genome. The assembly-activating protein (AAP), VP1, 2, and 3 are all translated from the p40 transcript encoded by the cap gene. Recombination is possible between AAV vectors carrying DNA sequences that are homologous to a particular chromosomal site and the corresponding genomic locus. loxP (locus of X-over P1) is a 34-bp site located on the bacteriophage P1. The site comprises an 8-base asymmetric sequence, variable except for the middle two bases, situated between two sets of 13-base symmetric sequences. The bacteriophage P1 life cycle depends on LoxP sites, which are essential for phage genome integrity and facilitating phage integration into the bacterial chromosome at the loxB target site (approximately every 90 kbp) [23–26]

Being a dependoparvovirus, AAV does not possess the necessary genes required for replicating and expressing its genetic material. These critical functions are facilitated by the adenovirus (Ad) E1, E2a, E4, and VA RNA genes [17, 18]. The genome of AAV consists of a singular-stranded DNA configuration, accommodating four recognized open reading frames (ORFs). The initial ORF encodes the four replication genes (Rep), named based on their molecular masses: Rep40, Rep52, Rep68, and Rep78 [19]. The following ORF is the Cap gene responsible for coding the trio of viral capsid proteins: VP1, VP2, and VP3. These proteins combine in a ratio of 1:1:10 to construct the icosahedral protein capsid composed of 60 subunits in the virus [19]. The third and fourth ORFs consist of nested sub-genomic mRNAs, with the third one named the assembly-activating protein (AAP). The AAP is involved in the transportation of capsid monomers to the nucleolus, where the process of capsid assembly takes place [20]; there is also the recently recognized membrane-associated accessory protein (MAAP), whose precise function has not been fully elucidated yet [21]. The 4.7-kilobase genome is bordered by 145-nucleotide inverted terminal repeats (ITRs) at both extremities of the genetic material (Fig. 1). The ITRs act as self-priming structures throughout the process of replication and also function as the signal for packing mediated by the Rep [22].

A total of 13 AAV serotypes (AAV1 to AAV13) exist, along with numerous additional variants originating from humans, other primates, and various mammalian species. These variants have been modified and utilized as vectors [27]. Furthermore, an array of chimeric capsids has been chosen from DNA libraries or strategically engineered, displaying improved attributes and progressing to clinical trials. Presently, over 100 AAV isolates have been discovered. Within the collection of human and non-human primate (NHP) AAVs, 13 serotypes have been documented and grouped into six phylogenetic clades, determined by their VP sequences and antigenic reactivities. Notably, AAV4, AAV5, AAV11, and AAV12 are clonal isolates within this classification. AAV1 and AAV6, falling under clade A, exhibit six disparities among 736 VP1 amino acids (with five amino acids in VP3), rendering them antigenically cross-reactive. Additional representatives from various clades contain AAV2 (clade B), the AAV2-AAV3 hybrid (clade C), AAV7 (clade D), AAV8 and AAV10 (clade E), and AAV9 (clade F) [28].

AAVs exhibit distinct preferences for particular organs and tissues in the body based on their serotype. Various AAV serotypes display differences in multiple aspects being investigated within the context of HCC. Research in this area has predominantly centered around subcutaneous xenograft models, with a notable focus on AAV2, AAV3, AAV6, AAV8, and AAV9 serotypes [29–32]. Subsequently, each serotype will be individually addressed and examined in detail.

### AAV2

AAV2 is the most-studied serotype. Initially identified in 1965, it emerged as an unintended presence during the preparation of simian Ad [33]. Nonetheless, the primary receptor of AAV2 alone was inadequate for efficient cell entry. Consequently, several co-receptors were identified, including αVβ5 and α5β1 integrins, laminin receptor (LamR), hepatocyte growth factor receptor (HGFR), human fibroblast growth factor receptor 1 (FGFR1), and CD9 [34]. Recombinant AAV2 capsids undergo diverse post-translational modifications (PTMs), encompassing ubiquitination, SUMOylation, phosphorylation, and multi-site glycosylation [35]. Expression cassettes that have been selected are encapsulated within distinct AAV capsids that target distinct tissues. Conventional capsids are typically derived from primates and humans, among other natural sources. Historically, among the eleven primary serotypes (AAV1 to AAV11) that have been cloned to date, AAV2 has been the most thoroughly characterized. It is widely believed that its packaging and transduction capabilities are sufficiently safe and efficient. Pseudotyped vectors, which alter the tissue tropism of a vector by encapsulating ITRs of one serotype in the capsid of another serotype, frequently employ it as the structural backbone [36]. The production and evaluation of AAV variant libraries have become a potent technique for discovering novel capsids that can be utilized in GT. Vast population diversity necessitates multiplexed production for libraries; this process involves transfecting cells containing a collection of ITR-containing plasmid variants to generate the viral library. The efficacy of this procedure may be compromised by cross-packaging and mosaicism, which occur when particles consist of capsid monomers and genomes sourced from various members of the library. The prevalence of cross-packaging and mosaicism in simplified, minimal libraries is investigated in a study by researchers employing novel assays that are specifically designed to evaluate capsid composition and packaging [37]. The phenomena of mosaicism and cross-packaging (also known as cross-typing) within AAVs have facilitated the encapsulation of the viral genome from one serotype into the capsid of another. AAV-2 vectors' extensive tissue tropism constituted a significant safety and specificity drawback, as transduction into non-target tissues was possible after vector administration. Cross-packaging of AAV-2 vectors onto distinct serotypes has been shown in recent research to increase transduction efficiency. Compared to AAV-2, cross-packaged AAV-2 genomes in AAV-1, AAV-3, and AAV-4 capsids increased gene expression in skeletal muscle by 900, 30, and 3 times, respectively. AAV-9 demonstrates a comparable profile to AAV-2 regarding broadly disseminated transduction, although it does so with a significantly higher efficacy. Therefore, the utilization of an AAV-2 vectors containing an AAV-9 capsid might potentially induce sustained transduction within cells. An additional approach to enhance specificity involves selecting a promoter that intrinsically stimulates the expression of a designated gene within the target tissue [38]. Antigenicity and tissue tropism are characteristics of viral particles determined by the capsid gene. Despite significant homology and identity in their capsid genes, the infectivities of AAV1 and AAV2 in muscle are notably dissimilar. Smooth muscle, skeletal muscle, the CNS, the liver, and the kidney are all targets of AAV2 tropism [39]. AAV2 additionally demonstrates a remarkable affinity for targeting hepatocytes [40]. AAVs have garnered increased attention as vectors for therapeutic gene delivery in light of recent clinical successes in GT applications. While prototypical AAV2 has demonstrated efficient transduction of human hepatocyte-derived cell lines in vitro, it has yet to be converted into an effective vector for GT targeting the liver in vivo. These results align with those obtained from Fah − / − /Rag2 − / − /Il2rg − / − (FRG) mice transfused with human livers, indicating that AAV2 exhibits suboptimal functionality in this xenograft model. Investigators demonstrated that naturally hepatotropic AAV capsid sequences were extracted from primary human liver samples. Capsid mutations, which were likely acquired inadvertently during tissue culture propagation, were shown to reduce the intrinsic hepatic tropism of natural AAV2 and related human liver AAV isolates, according to research. The amino acid modifications brought about by these mutations enhanced the affinity for heparan sulfate proteoglycan (HSPG), the principal cellular receptor that facilitates AAV2 infection of human hepatocytes. To facilitate AAV2 attachment, the capsid residues R484, R487, K532, R585, and R588 interact with the negatively charged polysaccharide chain of heparan sulfate. The tissue culture adaptation observed during in vitro propagation of natural AAV variants led to a reduction in tropism for human hepatocytes. By reducing AAV2's binding to HSPG, in vivo readaptation of the prototypical AAV2 in FRG mice with a humanized liver restored AAV2's intrinsic hepatic tropism. The findings of this study refute the hypothesis that AAV2 entry into human hepatocytes requires a high affinity for HSPG. Instead, they indicate that natural AAV capsids originating from the human liver may be more productive than culture-adapted AAV2 for liver-targeted GT applications [41, 42].

### AAV3

The most effective method for transducing primary human hepatocytes in vitro and "humanized" mice in vivo is via AAV serotype 3 (AAV3) vectors. This suggests that AAV3 vectors expressing human coagulation factor IX (hFIX) could potentially serve as a more efficient alternative for clinical GT of hemophilia B. Researchers expanded on these discoveries in the current work to create an AAV3 vector with an optimized hFIX cDNA sequence sandwiched between two AAV3 ITRs and a small yet potent liver-directed promoter. This vector produces therapeutic amounts of hFIX in vivo in hemophilia B patients and in "humanized" mice when enclosed in an AAV3 capsid. Together, investigations have produced an AAV3 vector that is anticipated to be clinically effective for hemophilia B GT at lower viral dosages without requiring immune suppression [43]. Like AAV2, AAV3, which has been isolated from humans, utilizes HSPG, FGFR1, LamR, and human HGFR (hHGFR) as its receptors [44, 45]. Glycosylation, phosphorylation, and acetylation are PTMs on rAAV3 capsids [35]. Initially, AAV3 was primarily overlooked for GT owing to its limited transduction capability in murine cell lines and in vitro. However, subsequent research revealed that utilizing hHGFR as a co-receptor markedly enhanced the transduction of human liver cancer cells, as well as NHP and human hepatocytes [46]. Indeed, AAV3 has demonstrated exceptional efficiency in transducing primary human hepatocytes, surpassing other serotypes in this aspect [47]. After identifying AAV3's distinct tropism, much research has been dedicated to improving the transduction efficacy of rAAV3 vectors. These efforts have included optimizing AAV3 vectors through capsid modifications, augmenting hHGFR expression, and modifying tyrosine kinase function [13, 48].

### AAV6

AAV6 shows considerable genetic resemblance to AAV1 and AAV2, yet it has been granted a distinct serotype designation. AAV6 has a serological signature similar to AAV1, a 99% homology coding area, and several sections comparable to AAV2. Thus, it was hypothesized to be a naturally occurring hybrid emerging from homologous recombination between AAV1 and AAV2. AAV6 was discovered in a human Ad sample, and like AAV1, it attached to sialylated proteoglycans, mainly sialic acid with α2,3 or α2,6 linkages, as its main receptor. It is also bound to heparan sulfate [49–51]. Epidermal growth factor receptor (EGFR) is its co-receptor [52]. The only observed PTM on rAAV6 capsid protein is acetylation [35].

DCs play a crucial role in regulating the adaptive immune response as antigen-presenting cells (APCs). Since antigen presentation is DCs' significant role and only DCs can trigger a primary immune response in resting naive T lymphocytes, DCs are special APCs and have been called "professional" APCs. Furthermore, considerable research has been devoted to investigating genetically modified DCs, and a multitude of Phase I and II clinical trials have been initiated to assess the effectiveness of DCs in carcinoma patients. It has been demonstrated that various serotypes of AAV vectors can successfully transduce distinct subsets of DCs; furthermore, the potential benefits of an AAV-based antitumor vaccine are discussed. To accomplish a substantial impact as an anti-tumor vaccine, however, additional enhancements in the specificity and transduction efficacy of gene transfer by recombinant AAV vectors to DCs are necessary [53, 54]. There has been a lot of interest in rAAVs as a possible vaccine because of their ability to transduce DCs and elicit T-cell response. Here, we demonstrate that among all the serotypes and variations of rAAV2, the pseudotype with the type 6 capsid (rAAV2/6) has the highest tropism for human monocyte-derived dendritic cells (MoDCs). A single lysine-to-alanine change inside the AAV6 capsid was demonstrated to hinder binding to heparin, and this alteration completely stopped transduction. In contrast to rAAV2, soluble heparin did not impede the transduction of MoDCs by rAAV2/6. An additional augmentation of MoDC transduction was noted after substituting Tyr-731 in the capsid of AAV6, which aligns with the notion that phosphorylation of tyrosine residues results in the ubiquitination of capsids during uptake. The immunophenotype of MoDCs was only marginally modified by pseudotyped rAAV2/6 vectors carrying a Y731F mutation; these cells retained their capacity to stimulate an antigen-specific CD8+ T cell clone. The discoveries should contribute to the advancement of rAAV2/6 as a vaccine vector [55]. In addition, rAAV6 is a more effective serotype for transducing human DCs and may respond to Hep3b cells [15, 55, 56]. By site-directed mutagenesis of surface-exposed serine (S) and threonine (T) residues, which play a crucial role in intracellular trafficking of AAV vectors, a capsid-optimized AAV6 vector (AAV6-T492V+S663V) was generated. Compared to wild-type (WT) AAV6 vectors, this double-mutant AAV6 vector exhibited a transduction efficiency in monocyte-derived DCs (moDCs) that was approximately five times greater. The enhanced nuclear translocation of AAV6-T492V+S663V was found to be correlated with the increased transduction efficiency in comparison to the WT-AAV6 vector. Further investigations into the CD11c promoter uncovered pivotal regulatory components that are compatible with the AAV expression cassette and stimulate the expression of EGFP in moDCs. EGFP expression in moDCs was significantly increased by developing a chimeric promoter (chmCD11c) comprising functional modules of CD11c and an enhancer element derived from the Simian virus (SV40). MoDCs, which were transduced using a capsid-optimized AAV6 vector carrying human prostate-specific antigen (hPSA) and CBA (AAV6-T492V+S663V-CBA-hPSA) or chmCD11c (AAV6-T492V+S663V-chmCD11c-hPSA), produced a more significant number of cytotoxic T lymphocytes (CTLs) with enhanced cytotoxic capabilities against human prostate adenocarcinoma cells (LNCaP) in comparison to CTLs induced with wild-type AAV6. These studies collectively indicate that optimizing the capsid and promoter regions of AAV vectors could potentially serve as a viable strategy to target molecular-disrupting cells (MDCs) efficiently and could develop into a promising instrument for cancer immunotherapy [15].

### AAV8

AAV8, similar to AAV7, was discovered in Rhesus macaque monkeys in 2002. AAV8 shares the same primary receptor as AAV2 and AAV3, the LamR [45, 57]. rAAV8 capsid proteins are phosphorylated, glycosylated, and acetylated as a matter of PTMs [35]. Serotypes AAV2, AAV5, AAV8, and AAV9 are commonly employed for transfecting liver cells. Research has indicated that AAV8 and AAV9 exhibit a notable affinity for liver cells, with AAV8 demonstrating the highest degree of hepatophilia. rAAV8 can transfect hepatocytes in primates, canines, and rodents with high efficiency and stability through intraperitoneal injection, portal vein, or peripheral vein. Research has shown that the expression of target genes mediated by AAV8 was approximately 10- to 100-fold greater in the liver than other serotypes [58]. This potency surpasses almost all different AAV serotypes across various models, encompassing mice, canines, and NHPs [59–61]. Proteasome inhibitors are a class of small molecule compounds designed to obstruct the proteasome's activity selectively. This leads to an accumulation of ubiquitinated proteins, an upregulation of intracellular reactive oxygen species, and an overall reduction in the presentation of MHCI–peptide complexes. Protein degradation mechanisms within cells are targeted by proteasome inhibitors, which function by impeding the β proteolytic subunits of the 20s proteasome. Their primary cytotoxic mechanisms include activating apoptotic pathways, inhibiting cell survival pathways, and increasing ER stress [62]. Protease inhibitors have been demonstrated to impact AAV transduction both in vitro and in vivo significantly. Research on polarized lung airway epithelia revealed that the co-application of tri-peptide proteasome inhibitors (LLnL) and AAV2 to the apical surface increased expression levels by more than 200-fold. Co-administration of tri-peptide proteasome inhibitors (LLnL or z-LLL) to the lung or liver resulted in a notable increase in expression. Nevertheless, no such enhancement was detected in skeletal or cardiac muscle. The observed effect of proteasome inhibitors to enhance AAV transduction has been documented across various serotypes and cell types [63]. Applying proteasome blockers can potentially improve AAV8 transduction rates in particular tissues [64]. In a study, researchers demonstrate that AAV-7 and -8 also exhibit limited efficiency in transducing endothelial cells and that inhibiting the proteasome significantly increases the levels of transgene expression. Proteasome inhibition increases the nuclear translocation of virions in both instances. Researchers additionally demonstrate that this is selective for vascular cell types, as proteasome inhibition does not affect the transduction of smooth muscle cells. These results were further supported by analysis in intact blood vessels, which indicates that degradation of proteasomes is a prevalent factor impeding the transduction of endothelial cells by AAV vectors [64].

### AAV9

AAV9 was initially identified in a human sample in 2004 and characterized as a novel serotype due to its distinct serological attributes compared to existing AAVs. However, subsequent investigations revealed its close association with clades containing AAV7 and AAV8 [65]. The principal receptor for AAV9 is the terminal N-linked galactose. Additionally, AAV9 employs the LamR and a potential integrin as co-receptors [66, 67]. The capsid of rAAV9 exhibits the most diverse range of PTMs, encompassing acetylation, ubiquitination, phosphorylation, SUMOylation, and glycosylation [35]. In most tissues, AAV9 demonstrates superior cell transduction capabilities compared to other AAV variants. In a mouse model involving systemic administration of AAV1–9, AAV9 exhibited the swiftest initiation of action, optimal dispersion of its genome, and the highest protein expression levels [68]. Utilizing a human liver chimeric mouse model (Table 1), researchers discovered that AAV9 holds promise as a viable candidate for gene transfer within human livers as well [69].

**Table 1:** The inception of prominent AAV isolates within clinical applications of liver-specific GT, encompassing their receptor affinities and tissue selectivity

Serotype	Origin	Receptor and co-receptors	Tissue tropism	References
AAV2	Human	HSPG, integrin αVβ5 and α5β1, HGFR, LamR, FGFR1, CD9, AAVR	Liver, SM, CNS, Kidney, Retina Heart, Lungs	[45, 70–72]
AAV3	Human	HSPG, LamR, FGFR1, HGFR, AAVR	Liver, HCC, SM, cochlear inner hair cells	[45, 70–73]
AAV6	Human	α2,3/ α2,6 N-linked SA, EGFR, HSPG, AAVR	Liver, SM, Heart, Airway, Retina	[45, 70, 71]
AAV8	NHP	LamR, AAVR	Liver, Retina, SM, CNS, Pancreas, Kidney, Heart	[45, 70, 71]
AAV9	Human	N-linked galactose, LamR, AAVR	Liver, Heart, SM, Pancreas, CNS, Retina, Lung,	[45, 70, 71]

## Oncolytic and recombinant AAV (rAAV)

In rAAV, the Rep and Cap genes are excised and replaced with the transgene expression unit. As a result, the sole viral genome sequences that endure are the ITRs, which play a crucial role in packaging the vector genome [27]. The processing of rAAV vector genomes depends on the cellular machinery of the host, including DNA repair systems, to enable consistent transduction. Upon AAV vector transduction, the single-stranded rAAV vector genomes are blueprints for forming double-stranded (ds) linear rAAV monomers [16]. Subsequently, these ds linear monomers progress to create ds circular monomers and DNA concatemers under the guidance of host DNA polymerases and DNA repair pathways. Wild-type (WT) AAV can be sustained either as an episome or can integrate into the genome of the target cell. This integration process is facilitated by Rep, which has a preference for a specific locus on chromosome 19 in humans. Integration can also occur at various other chromosome sites [74]. In contrast to WT AAV, the DNA of rAAV lacks the active integration process facilitated by Rep and predominantly maintains a circular concatemeric episomal configuration. However, rAAV vectors exhibit a low-frequency integration into the genome of target cells, a phenomenon potentially guided by endogenous enzymes altering host cell DNA. This integration mechanism raises concerns regarding the potential for genotoxicity resulting from rAAV genome integration [75].

**Fig. 2:**
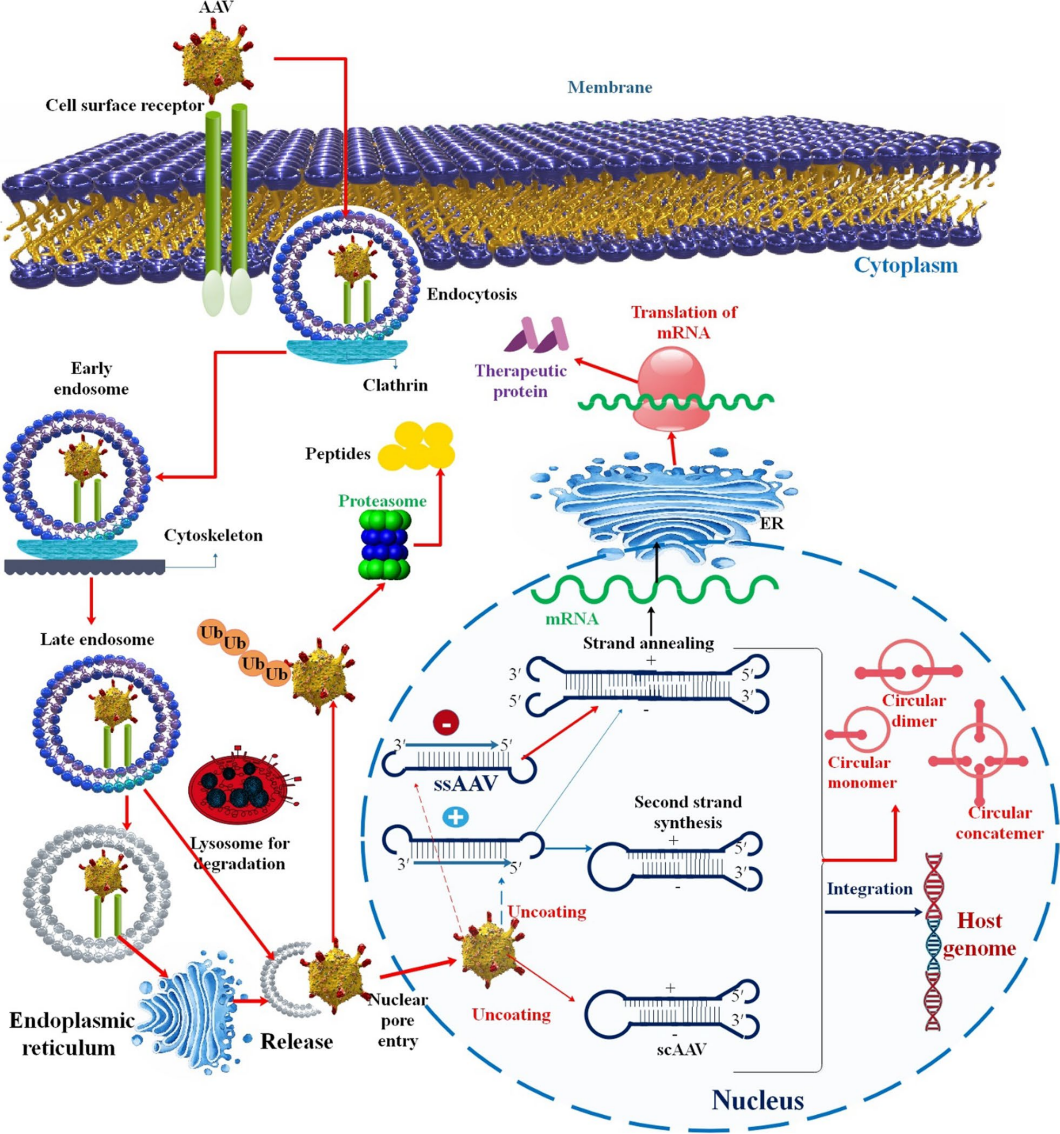
Diagram of rAAv transduction pathway. Adeno-associated virus (AAV) is recognized by glycosylated cell surface receptors of the host. This triggers the internalization of the virus via clathrin-mediated endocytosis. AAV then traffics through the cytosol mediated by the cytoskeletal network. Owing to the somewhat low pH environment of the endosome, the VP1/VP2 region undergoes a conformational change. Following endosomal escape, AAV undergoes transport into the nucleus and uncoating. AAV can also undergo proteolysis by the proteasome. There are currently two classes of recombinant AAVs (rAAVs) in use: single-stranded AAV (ssAAV) and self-complementary AAV (scAAV). ssAAVs are packaged as either sense (plus-stranded) or anti-sense (minus-stranded) genomes. These single-stranded forms are still transcriptionally inert when they reach the nucleus and must be converted to double-stranded DNA as a prerequisite for transcription. This conversion can be achieved by second-strand synthesis via host cell DNA polymerases or by annealing the plus and minus strands that may coexist in the nucleus. Because scAAVs are already double-stranded by design, they can immediately undergo transcription. The viral ITRs present in the rAAV genome can drive inter-molecular or intra-molecular recombination to form circularized episomal genomes that can persist in the nucleus [16]

Oncolytic viruses (OVs) constitute a novel category of cancer treatments that foster tumor regression by selectively replicating within tumor cells. They achieve this through inducing immunogenic cell death and activating the host's antitumor immune response. Currently, four OVs have received global approval for treating advanced cancers like melanoma, nasopharyngeal carcinoma, and glioblastoma [76, 77]. These findings have prompted a transition in utilizing OVs, shifting them from solely lytic agents to now serving as agents that stimulate the antitumor immune response. Consequently, the field is now more accurately called "oncolytic immunotherapy." Another emerging dimension of OVs is their capacity for combining conventional and contemporary cancer treatment methods, notably immune checkpoint inhibitors (ICIs) and medicines centered around T cells [78, 79]. The success of oncolytic virotherapy and GT hinges on the proper conveyance of the viral vector and precise targeting of the intended tissues. The main challenge that often diminishes the efficacy of these therapies is the inadequate transduction of the target tissues, leading to insufficient expression of the therapeutic transgene [80]. An optimal candidate for an oncolytic virus should exhibit specific characteristics, including a thorough comprehension of its biology and genetics. The chosen oncolytic virus should be immunogenic, able to induce cell lysis in infected cancer cells, and devoid of the potential to cause chronic infections or infectious diseases. Additionally, it should lack the capability to be incorporated into the human genome [81]. For example, by containing tissue-specific promoters, the expression of the vector's payload can be more finely restricted to specific cell types (Fig. 2). Specifically, integrating the Cre recombinase alongside a hepatocyte-specific promoter, such as the Thyroxin Binding Globulin (TBG) promoter, into the AAV8 genome can offer a targeted strategy for expressing Cre recombinase within hepatocytes. This approach has been demonstrated to impact the cell cycle of hepatocellular cells and trigger a DNA damage response, all while preventing unintended expression in non-hepatic cells [82, 83]. Currently, clinical trials have indicated that oncolytic AAV therapy leads to minimal adverse events, underscoring a favorable safety profile for this approach [84].

### Examples of oncolytic AAV and rAAV in development

#### rAAV-GSDMDNT (rAAV-P1) and rAAV-DIO-GSDMD^NT^ (rAAV-P2)

Lu et al. explored the potential of rAAV-P1 and rAAV-P2 to induce pyroptosis in tumor cells, particularly in the Hep3B cancer cell line. Both rAAVs effectively triggered pyroptosis by expressing the N-terminal gasdermin domain (GSDM^NT^), extending lifespan in preclinical cancer models. The OVs not only initiated pyroptosis but also amplified the immune response. The study highlighted the synergistic effect of combining OVs with anti-PD-L1 therapy, presenting a promising avenue for enhanced anti-tumor strategies [85].

## AAV-3-S663V+T492V-Trichosanthin (TCS, a ribosome-inactivating protein)

rAAV3 vectors exhibit efficient liver cancer cell targeting in vivo, with enhanced transduction efficiency upon removal of specific surface-exposed serine and threonine residues on capsids. This adjustment upholds viral tropism and the binding to cellular receptors. Ling et al. uncovered the dual functionality of shikonin, acting as a liver tumor growth inhibitor and an enhancer of rAAV vector-driven GT effectiveness in vivo. The researchers successfully suppressed tumorigenesis in a liver cancer xenograft model by applying enhanced rAAV3 vectors and shikonin administration, both individually and synergistically. The integration of rAAV3-derived GT and chemotherapy with shikonin, involving the use of rAAV3−S663V+T492V-TCS vectors at a dosage of 5 × 10^10 vg/mouse and administering shikonin at 1 mg/kg/day for five sequential days, resulted in significant suppression of tumor growth. This combination demonstrates a promising approach for highly effective anti-tumor intervention [86, 87].

## AAV in gene therapy of HCC

According to the American Society of Gene and Cell Therapy (ASGCT), as of their latest definition in 2019, GT is described as "the introduction, removal, or modification of a person's genetic code to treat or cure a disease." This definition encompasses a range of GT approaches, including conventional methods like gene addition or supplementation, as well as gene editing approaches that involve modifying, repairing, or introducing DNA sequences into the cellular genome. Furthermore, it encompasses gene silencing methods via RNA interference or precise nuclease targeting [88]. In the effort to address both localized and advanced liver cancer, multiple vector systems and strategies are presently being devised. One notable system in this arena is the AAV vectors. AAV vectors have gained significant prominence, with over 405 human clinical trials and three GT drugs approved [89, 90]. Indeed, AAV vectors have been employed in advanced stages of human clinical trials for addressing monogenetic liver-based diseases including hemophilia A and B. In 2022, significant milestones were achieved in this regard. The European Medicines Agency has provided provisional approval for valoctocogene roxaparvovec (AAV5-hFVIII-SQ), an AAV5-derived GT developed to address hemophilia A [91]. Additionally, Etranacogene dezaparvovec-drlb (AAV5-FIX Padua) has received approval from the FDA for treating hemophilia B [92].

Indeed, AAV vectors have been modified to treat individuals suffering from liver disorders such as viral hepatitis, familial hypercholesterolemia, and liver cancers. A noteworthy early experiment in the context of GT for HCC involved the application of AAV vectors conducted by Su et al. In this study, a recombinant AAV virus was engineered, containing the herpes simplex virus thymidine kinase (TK) gene. This gene was regulated by the human α-fetoprotein (AFP) enhancer and the albumin promoter. The result was a targeted cytotoxic impact on HCC cells that expressed AFP while leaving non-hepatocyte tumor cells and hepatic tumor cells lacking AFP and albumin unaffected [93].

**Fig. 3:**
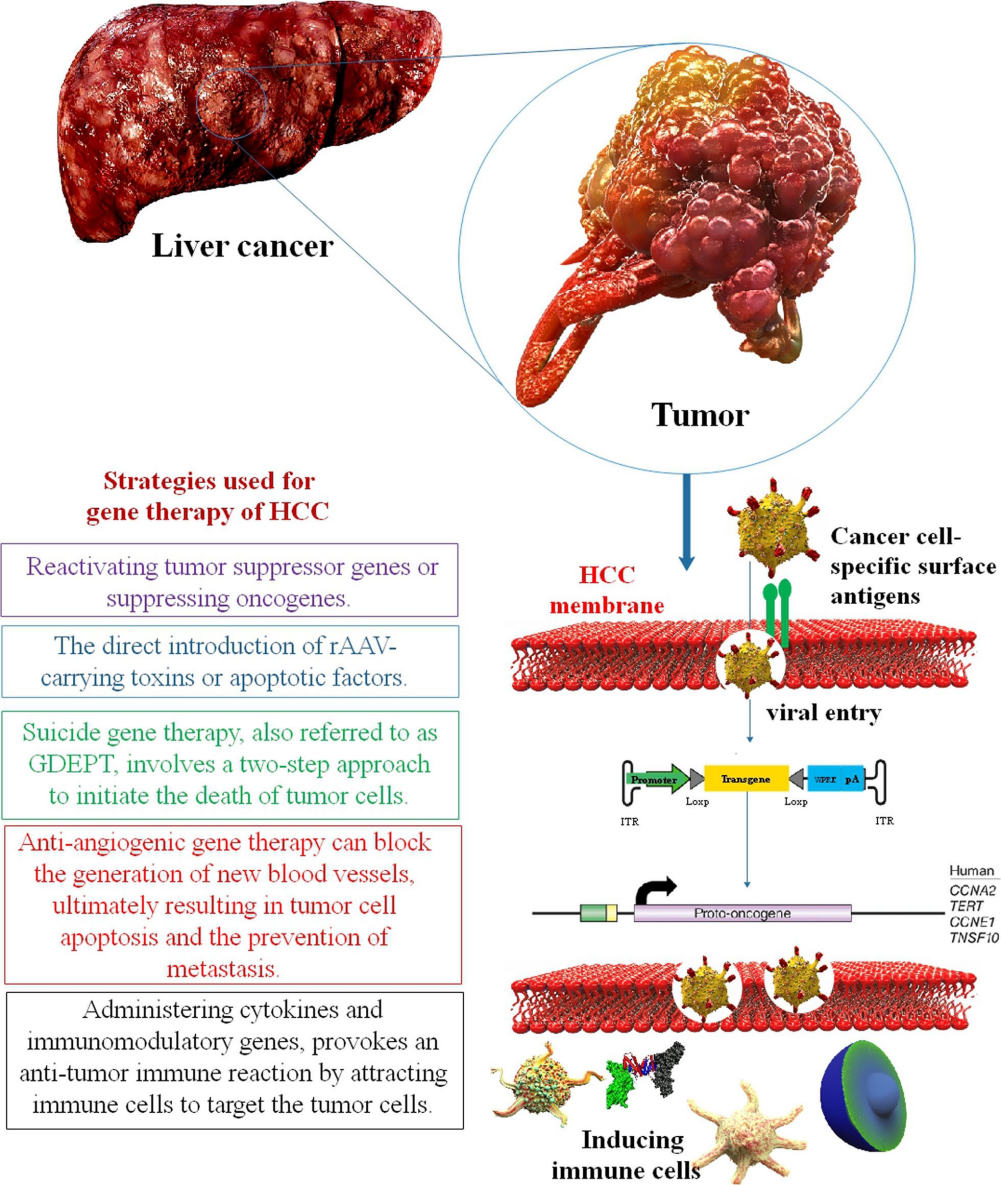
Strategies used for gene therapy of HCC

Dhungel et al. categorized the approaches employed for GT of HCC, drawing upon a compilation of preclinical research involving AAV vector testing, which is as follows: (1) Reactivating tumor suppressor genes or suppressing oncogenes can potentially reinstate the normal functionality of cancerous cells. (2) Directly introducing rAAV-carrying toxins or apoptotic factors such as TRAIL can induce cytotoxicity and/or trigger apoptosis in tumor cells. (3) Suicide GT, also referred to as GDEPT (Gene-Directed Enzyme/Prodrug Therapy), involves a two-step approach to initiate the death of tumor cells. Initially, tumor cells are genetically altered using rAAV to express a suicide gene. Subsequently, a prodrug is administered systemically, which then undergoes metabolic conversion within the transduced cells into a toxic compound, ultimately leading to cell death. (4) Utilizing AAV vectors for anti-angiogenic GT can block the generation of new blood vessels, ultimately resulting in tumor cell apoptosis and the prevention of metastasis. (5) Administering cytokines and immunomodulatory genes, whether through AAV vectors directly or via immune cells modified with rAAV vectors carrying cytokines (adopted immunotherapy), provokes an anti-tumor immune reaction by attracting immune cells to target the tumor cells [94]. The fundamental concepts of each of these approaches have been depicted in (Fig. 3), and we will proceed to provide novel instances for each one, with the clarification that some cases may be a combination of multiple strategies.

### Reviving tumor suppressor genes/Blocking oncogenes

Since AAVs lack replication capabilities, they offer a relatively safe and effective method for expressing the Cre recombinase, overexpressing particular proteins, or introducing shRNA into in vivo model systems [95, 96]. As an alternative to employing Cre recombinase, various constructs can be utilized, such as those facilitating shRNA expression or the introduction of external proteins to target hepatocytes via tissue-specific promoters. For instance, the AAV8-TBG-P21 vector can achieve hepatocyte-specific overexpression of P21, effectively curbing their proliferation [97]. AAV vectors have been documented to sustain the expression of ectopic proteins for an extended period, often lasting several months, particularly in post-mitotic cells [98]. Furthermore, RNA interference (RNAi) strategies, including antisense targeting of hypoxia-inducible factor-1α and microRNA (miRNA)-focused therapies facilitated by AAV, have been utilized as anti-cancer treatments for HCC [99, 100]. In addition, the systemic delivery of miR-26a through AAV vectors effectively restrained HCC cell proliferation, prompted apoptosis specifically in tumor cells, and curbed tumor formation in a mouse model of liver cancer [100]. Alongside the conventional approach for miRNA replacement therapies, a method to inhibit the oncogenic miR-221 using miRNA sponges has been devised for HCC therapy. In this approach, AAVs were genetically engineered to induce the expression of several sites for miR-221 binding [101]. Combination therapy plays a crucial role in clinical cancer treatment. AAV-mediated GT is a promising complement to other therapeutic approaches.

*AAV-3-miRNA*: As AAV3 vectors exhibit superior transduction of human hepatic cells compared to AAV8 vectors, Yin et al. assessed AAV3-miR-26a/122 vectors' efficacy in inhibiting human HCC cells in vitro and liver tumors in mice in vivo. Given the superior transduction efficiency of AAV3 vectors over AAV8 vectors by human hepatic cells, the objective of the current investigations was to assess the effectiveness of AAV3-miR-26a/122 vectors in inhibiting the proliferation of HCC cells in vitro and murine models of human liver tumors in vivo. To accomplish this, different multiplicities of infection (MOIs) of AAV3-miR-26a, scAAV3-miR-122, or both vectors co-expressed a Gaussia luciferase (GLuc) reporter gene onto the human HCC cell line Huh7. At the maximum molecular weight (MOI) of 1 × 10^5^ vgs/cell, only a moderate degree of growth inhibition (12–13%) of Huh7 cells was detected dose-dependent with each vector. A growth inhibition of approximately 26% was observed when Huh7 cells were co-transduced with both vectors. On the other hand, in vivo, mouse xenograft models, AAV3-miR-26a, and scAAV3-miR-122 vectors inhibited the growth of Huh-derived human liver tumors by approximately 70%. Therefore, the potential utility of employing scAAV3-miR-122 and miR-26a delivered via AAV3 vectors in conjunction to target human liver tumors is demonstrated [102].

*AAV-8-NPC2*: NPC2 is typically found in high levels in healthy liver tissue but experiences downregulation in human HCC tissues. Knocking down NPC2 in liver cancer cell lines has been observed to stimulate cell proliferation, migration, and the formation of xenograft tumors. Conversely, when NPC2 is overexpressed, it inhibits tumor growth promoted by HuH7 cells. Moreover, the delivery of NPC2 via hepatotropic AAV8 has been shown to reduce inflammatory infiltration, decrease the expression of two early HCC indicators (survivin and glypican 3), and suppress the HCC onset occurring spontaneously in mice [103].

### Delivery of toxins and pro-apoptotic factors

TNF-related apoptosis-inducing ligand (TRAIL) can trigger apoptosis, or programmed cell death, in a broad range of tumor cells while sparing most normal cells. Nevertheless, it has become evident that numerous primary cancer cells exhibit resistance to TRAIL treatment when used as a standalone therapy [104].

Ma et al. discovered that the miR-221/222 cluster is overexpressed in liver cancer cells that exhibit resistance to apoptosis triggered by TRAIL, promoting cell proliferation and inhibiting apoptosis. They developed miR-Zip inhibitors targeting miR-221/222 and used AAV-mediated GT to co-express TRAIL and miR-221-Zip, which enhanced cell death in vitro. In vivo experiments on mice with liver cancer xenografts displaying resistance to TRAIL treated with AAV-TRAIL-miR-221-Zip showed suppression of tumor growth [105].

Wang et al. conducted a study to explore the potential of combining AAV-hTERT-TRAIL and cisplatin for treating HCC. They observed increased TRAIL expression in BEL7404 hepatoma cells treated with AAV-hTERT-TRAIL and cisplatin. The combined treatment exhibited higher cytotoxicity and induced more substantial cancer cell apoptosis than AAV-hTERT-TRAIL or cisplatin alone. In animal trials, this combination therapy effectively suppressed tumor growth and led to tumor cell death [106]. Additional research has demonstrated that radiotherapy can potentially augment the uptake of recombinant AAV in HCC cells in vitro and in vivo [107].

*AAV-9-shGαi2*: Chen et al. have identified Gαi2 (G protein subunit alpha i2) as a potential therapeutic target and diagnostic marker for HCC due to its overexpression in HCC tissues and cells, which is associated with a less favorable prognosis for patients. Their study demonstrated that silencing Gαi2 (by targeted shRNA) or knockout (by the dCas9-sgRNA method) significantly inhibited cell growth and migration, induced cell cycle arrest, and triggered apoptosis in HCC cells [108]. The number of Gαi2 transcripts in HCC tissues is considerably greater than in normal liver tissues, according to the Cancer Genome Atlas Liver HCC (TCGA-LIHC) database. Additionally, there exists a correlation between Gαi2 overexpression in HCC and unfavorable prognosis among patients. Additionally, both the mRNA and protein expression of Gαi2 are upregulated in various human HCC cells and local HCC tissues. Gαi2 silencing (via targeted shRNA) or knockout (KO, by the dCas9-sgRNA method) significantly inhibited cell proliferation and motility in immortalized HepG2 cells and patient-derived primary HCC cells. Additionally, it induced cell cycle arrest and caspase-apoptosis activation. Furthermore, reactive oxygen species (ROS) generation and oxidative damage were caused in primary and HepG2 HCC cells by Gαi2 silencing or KO. On the other hand, various antioxidants mitigated the anti-HCC cell activity induced by Gαi2-shRNA. Gαi2 overexpression was detected to enhance the proliferation and motility of primary and immortalized HCC cells via lentiviral construction. Subsequent research demonstrated a substantial increase in the binding affinity between early growth response zinc finger transcription factor 1 (EGR1), a transcription factor, and the Gαi2 DNA promoter in HCC cells and tissues. Intra-tumor injection of Gαi2 shRNA AAV in nude mice induced a substantial inhibition in the growth of HCC xenografts. Furthermore, the development of HCC xenografts in nude mice lacking Gαi2 was noticeably sluggish. Xenografts of HCC that were Gαi2-silenced or Gαi2-KO exhibited Gαi2-depletion, oxidative damage, and induction of apoptosis. HCC cell proliferation in vitro and in vivo depends on the overexpression of Gαi2. This characteristic establishes Gαi2 as an innovative and promising diagnostic marker and a therapeutic target for HCC [108].

### Suicide gene therapy, also recognized as GDEPT (Gene-Directed Enzyme/Prodrug Therapy)

Three primary systems have been widely employed in suicide GT: (1) Herpes Simplex Virus TK/Ganciclovir (HSV-TK/Ganciclovir). In this approach, the conversion of the GCV prodrug into its cytotoxic form, GCV-triphosphate, leads to the creation of a DNA chain terminator. When this terminator is integrated into DNA replication, it ultimately results in cell death. (2) The cytosine deaminase gene (CD) from Escherichia coli plays a role in transforming the pro-drug 5-Fluorocytosine (5-FC) into 5-Fluorouracil (5-FU). This process is one of the standard approaches employed in chemotherapy to treat HCC [5, 109]. (3) Purine nucleoside phosphorylase (PNP), an enzyme found in Escherichia coli, is responsible for converting the prodrug fludarabine phosphate (FP) into the active drug 2-fluoroadenine. When HCC cells were transfected with the PNP gene and treated with ultrasonic nanobubbles, they exhibited an elevated level of apoptosis when exposed to fludarabine phosphate. This system also demonstrated a significant bystander effect, mainly when prodrug concentrations were low [110]. Several bicistronic rAAVs were constructed to examine the impact of combined transduction of a suicide gene and genes encoding diverse immunostimulatory factors on the oncogenicity and immunogenicity of TC-1 cells (C57BL/6 mouse cells transformed with HPV-16). The herpes simplex type 1 thymidine-kinase gene (HSV-TK) and the gene of one of the subsequent immunostimulatory factors—human monocyte chemoattractant protein 1 (MCP-1), mouse B7.1 costimulatory molecule (B7.1), or mouse granulocyte–macrophage colony-stimulating factor—were carried by each of these constructs and expressed in infected cells. An rAAV carrying the neomycin resistance gene (neo) and the HSV-TK gene, as well as a rAAV carrying the lacZ gene, were utilized as controls. These constructs demonstrated functionality in human 293T cells and rodent TC-1 cells. To conduct experiments on mice, TC-1 cells were inoculated in vitro with AAV recombinants at a multiplicity of 50 particles per cell. Subsequently, 5-week-old mice were administered these cells. Half of the animals were administered ganciclovir (GCV) at a dosage of 2.5 mg per day for 10 days, commencing on day 5. With the sole exception of those inoculated with cells treated with rAAV expressing HSV-TK+B7.1 or HSV-TK+MCP-1, no tumors developed in the rodents, regardless of the administration of GCV. Animals inoculated with TC-1 cells infected with rAAV expressing HSV-TK+GM-CSF exhibited a diminished tumor suppressive effect. However, among these animals, the effect was marginally more pronounced in those not treated with GCV. GCV treatment exhibited a distinct antitumor effect exclusively in mice inoculated with TC-1 cells transduced with rAAV expressing HSV-TK; no immunostimulatory factor was detected. Tissue-free mice on day 54 were subjected challenged with untreated TC-1 cells. The observed rates of tumor resistance were found to be associated with GCV treatment in addition to the immunostimulatory gene used for transduction. Mice pre-inoculated with TC-1 cells transduced with B7.1 or MCP-1-expressing rAAV and not administered GCV exhibited the highest level of protection [111]. Due to its inherent hepatotropic nature, recombinant AAV2 is an optimal vector for suicide gene transfer in liver malignancies [112, 113]. Vexosomes, which are AAVs associated with exosomes, serve as an additional platform for gene delivery. Unexplored is the effectiveness of such vexosomes in suicide GT. Using a differential ultracentrifugation-based protocol, scientists produced AAV serotype 6 vexosomes containing an inducible caspase 9 (iCasp9) suicide gene in the current investigation. When primed with a pro-drug (AP20187), vexosomes containing AAV6-iCasp9 exhibited a substantial reduction in cell viability (57% ± 8% versus 100% ± 4.8%, *p* < 0.001) when compared to mock-treated Huh7 cells. When AAV6-iCasp9 vexosomes and AP20187 were administered intratumorally to murine xenograft models, tumor regression increased 2.3-fold compared to untreated animals. Additional confirmation of these results was obtained through histological examination and apoptosis assays. These results conclude that AAV6 vexosomes can be therapeutically utilized in a xenotransplantation model of HCC [30].

#### AAV-6-iCasp9

A disproportionately large quantity of vectors is liberated in the culture supernatant during recombinant AAV synthesis; this supernatant is frequently discarded. Research has demonstrated that these vectors often form associations with vesiculated entities, including exosomes. Vexosomes, which are AAVs associated with exosomes, serve as an additional platform for gene delivery. A fraction of AAV vectors associated with microvesicles/endo vesicles (exosome (exo)-associated AAVs or vexosomes) are naturally released into the supernatant fraction of the cell-culture media during vector production, according to a recent report. Regarding gene transfer to the retina, nervous system, and inner ear, these exo-AAV vectors exhibit superior performance compared to conventionally purified AAV vectors. Furthermore, exo-vectors are resistant to neutralizing antibodies and have a higher transduction efficiency, according to several studies. Particularly for therapeutic applications in vivo, where endogenous anti-AAV antibodies frequently compromise the therapeutic efficacy of gene delivery, the latter characteristic may be significant [30, 114, 115]. To turn off genes during T cell therapy, an inducible caspase 9 (iCasp9) gene has been evaluated. This gene is a synthetic analog of caspase 9 from mammals, based on mammalian caspase 9, fused to a human FK506 binding protein (FKBP). Conditional dimerization to an artificial, bioinert small molecule (chemical inducer of dimerization (CID), AP20187)) is possible. Except for a brief linker peptide sequence and a single amino acid substitution in the FKBP, iCasp9 is a self-protein and, therefore, non-immunogenic. Furthermore, since the suicide trigger is not influenced by the phase of the cell cycle, it may prove beneficial in the treatment of HCC, a chronic and slow-growing malignancy. Numerous investigations have demonstrated the specificity and effectiveness of iCasp9 in targeting cells [113]. A gene delivery vector containing the iCasp9 gene, a synthetic analog of mammalian caspase 9, was employed by researchers. This gene is fused to a human FK506 binding protein, which facilitates the conditional dimerization of the vector to a synthetic small molecule known as a chemical inducer of dimerization (AP20187). The iCasp9 induces apoptosis in the target cells. The potential anti-tumorigenic effect of these synthetic vectors based on an AAV platform was evaluated in vitro on human HCC cells and in a nude mouse model of HCC tumors in this investigation. The results indicate that the iCasp9-AP20187 bioconjugate can activate terminal effectors of cellular apoptosis, thereby establishing a feasible strategy for the prospective management of HCC [113]. In recent research on suicide GT for HCC, Khan et al. introduced an innovative approach using AAV6-based vexosomal vectors at minimal dosages (2 × 10^10 vgs). They developed AAV serotype 6 vexosomes encapsulating an inducible caspase 9 (iCasp9) suicide gene using a specialized ultracentrifugation-based technique. These vexosomes demonstrated significant cytotoxicity in vitro and in vivo human HCC models. When activated with the pro-drug AP20187, AAV6-iCasp9 vexosomes led to a notable reduction in cell viability (57% ± 8% compared to 100% ± 4.8% in mock-treated Huh7 cells, *p* < 0.001) in the in vitro model. When AAV6-iCasp9 vexosomes and AP20187 were administered intratumorally to murine xenograft models, tumor regression increased 2.3-fold compared to untreated animals. Additional confirmation of these results was obtained through histological examination and apoptosis assays. In summary, the therapeutic potential of AAV6 vexosomes in a xenotransplantation model of HCC is supported by our findings. Moreover, the ease of synthesis and isolation of vexosomes ought to enhance its potential for implementation in additional malignancies. [30].

### AAV vector-mediated targeting of tumor angiogenesis

The anti-angiogenic and anti-proliferative properties of Angiotensin-(1-7) have been confirmed in various cancer cells [116, 117]. In a study by Mao et al., they created an AAV8 capsid mutant (Y703F) that causes notably enhanced transgene expression on a systemic scale. This modification resulted in enduring and highly efficient expression of Ang-(1-7) in vivo, leading to a significant reduction in the growth of HCC. This effect was achieved by reducing the expression of pro-liferative and pro-angiogenic agents in a murine H22 hepatoma model. The removal of the Vascular Endothelial Growth Factor (VEGF) and Placental Growth Factor (PGF) further validated this outcome [118].

### Delivery of cytokines using AAV vectors to modify the immunosuppressive microenvironment

Employing cytokines as an adjunct in cancer immunotherapy presents a promising avenue [119]. Interferon-γ (IFN-γ) is a versatile cytokine with various roles, including antiviral, antitumor, and immunomodulatory functions [120]. Zhou et al. used an AAV2 vector to introduce IFN-γ expression using the Hep3B HCC cell line. Another AAV2 vector was employed to transduce DCs to express AFP, a tumor antigen associated with HCC. This led to a robust AFP-specific CTL response. The combination of AFP expression in DCs and the presence of IFN-γ from the AAV vectors, which resulted in increased expression of human Leukocyte Antigen A2 (HLA-A2) in Hep3B cells, heightened the responsiveness of CTL targeting AFP [56, 121]. Recent advancements have spotlighted the prospect of enhancing the safety and efficiency of T cell-based cancer therapy by engineering Chimeric Antigen Receptor-T (CAR-T) cells through Clustered regularly interspaced short palindromic repeat (CRISPR)-associated 9 (Cas9) nuclease (CRISPR/Cas9) gene editing. The accessibility and remarkable efficacy of CRISPR/Cas9 gene editing techniques have facilitated proficient gene knockout, precise site-directed gene insertion, and comprehensive genome-wide screening within T cells [122, 123]. CRISPR/Cas9-mediated cytokine modulation reduces toxicity and enhances the function of CAR T-cells. Implementing genetic approaches aimed at controlling cytokine signaling during the activation and expansion of CAR T-cells may result in improved antitumor efficacy, prolonged T-cell survival, and/or diminished toxicity [122]. An innovative approach involved using an AAV virus to express CCL19 in tandem with CAR-T cells that specifically target the GPC3 antigen, aiming to treat HCC both in vitro and in vivo. Findings have revealed that the introduction of CCL19 expression via AAV-CCL19 within tumor tissues facilitated the movement of memory T cells, such as memory CAR-T cells, into the core of the tumor. This resulted in an augmented quantity of CAR-T cells infiltrating the interior of the tumors, thereby leading to enhanced suppression of tumor growth [124]. A system was devised by researchers to generate chimeric antigen receptor (CAR)-engineered T cells (CAR-T cells) with significantly improved characteristics in an efficient manner through the implementation of streamlined genome engineering. Researchers utilized AAV to trans-activate CRISPR-independent CRISPR–Cpf1 systems, enabling the construction of a stable CAR-T cell that exhibited immune-checkpoint knockout and homology-directed repair (KIKO CAR-T cell) functionalities with a single-step efficiency. The AAV–Cpf1 KIKO system's modularity facilitates the generation of double knock-ins of two distinct CARs within a single T cell in a highly efficient and adaptable manner. Compared to methods utilizing Cas9, the efficiency of generating double-knock-in CAR-T cells is enhanced by the AAV–Cpf1 system. CD22-specific AAV–Cpf1 KIKO CAR-T cells exhibit similar cytokine secretion and cancer cell killing potency as Cas9 CAR-T cells despite expressing fewer exhaustion markers. This adaptable system effortlessly creates new T-cell engineering capabilities [125].

## Novel gene editing techniques combined with AAV for HCC

Recent progress in gene editing tools has expanded the potential for addressing various genetic disorders. Three prominent platforms that offer significant promise in this field are transcription activator-like effector nucleases (TALEN), zinc finger nucleases (ZFN), and CRISPR/Cas9 [126]. The remarkable versatility of CRISPR-Cas9 and TALENs in adapting to emerging genomic sequences has initiated a transformation in genome editing. This adaptability has played a pivotal role in accelerating scientific advancements across various disciplines, including human GT, disease modeling, synthetic biology, and drug development. ZFNs and TALENs share common features, including a FokI nuclease domain and a customizable set of motifs that can be programmed to identify particular DNA sequences for precise site-specific cleavage [127]. In contrast, the CRISPR/Cas9 system operates differently. It relies on a single guide RNA (sgRNA) that makes a complex with the Cas9 nuclease. This sgRNA is designed to recognize a specific 20-nucleotide target DNA sequence next to a protospacer adjacent motif (PAM). After it connects with the target DNA, the sgRNA-Cas9 complex induces a cut at the precise location specified by the sgRNA, enabling accurate genome editing [128]. ZFNs, TALENs, and CRISPR/Cas9 are all capable of efficiently inducing DNA double-strand breaks (DSBs) at particular pre-selected target sites. After the DSB is created, the cell's repair machinery can operate through two primary mechanisms: homology-directed repair (HDR) or non-homologous end joining (NHEJ). NHEJ frequently results in gene disruptions or mutations, while HDR can be harnessed for targeted gene integration or precise modifications [129, 130].

**Fig. 4:**
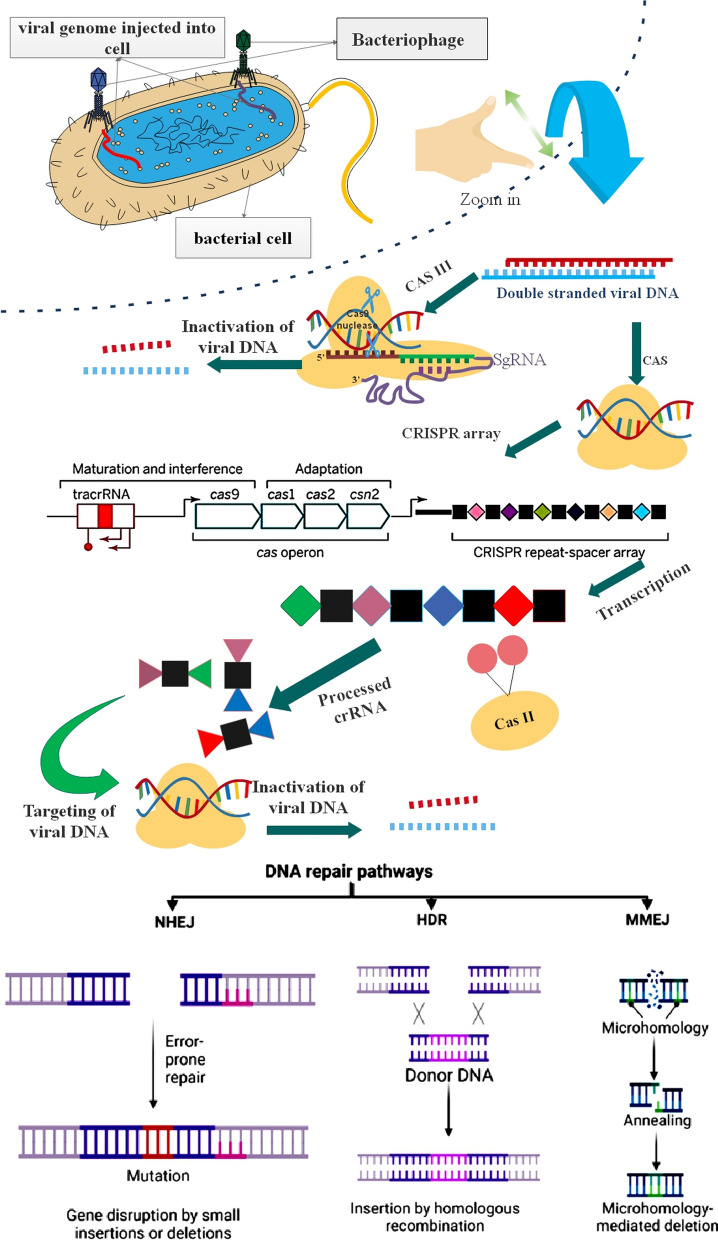
Major pathways involved in the mending of DNA damage after CRISPR/Cas-mediated DNA cleavage [133]. Microhomology-mediated end joining (MMEJ); Non-homologous end-joining (NHEJ); Homology-directed repair (HDR)

Indeed, CRISPR/Cas9 is the top choice for gene editing in recent times because of its simplicity, exceptional efficiency, and flexibility for customization [88]. NHEJ repair is error-prone and can lead to the disruption of a target gene's function through the initiating slight insertions or deletions (indels) at the cleavage point [131]. In contrast, the HDR pathway utilizes homologous DNA sequences to guide DNA repair by facilitating a strand-exchange process. This allows for the replacement of genome segments with donor DNA, relying on the presence of homologous sequences flanking the target site [132] (Fig. 4).

Moving forward, we will delve into recent advancements in Therapeutic approaches utilizing CRISPR technology and the delivery of CRISPR components through AAV vectors.

### The CRISPR/Cas system holds significant potential for gene therapy in liver cancer

CRISPR/Cas systems have been commonly employed in HCC treatment through two primary approaches: (1) direct editing of the intended targets and (2) targeting an indirect site to counteract the advancement of HCC. In direct targeting, genes linked to HCC, which encompass both oncogenes and tumor suppressor genes (TSGs), are the specific therapeutic targets. Zhu et al. employed CRISPR/Cas9 to disrupt the transcription factor ZIC2 in HCC cells, resulting in a remarkable suppression of tumor growth [134]. In indirect strategies, CRISPR-mediated gene manipulations are combined with immunotherapy, antitumor drugs, and different treatments or alterations to enhance their efficacy [135]. For example, a study demonstrated that inhibiting Extracellular signal-regulated kinase 2 (ERK2) kinases using CRISPR/Cas technology enhanced the responsiveness of HCC cells to sorafenib, a multi-kinase inhibitor widely prescribed for HCC treatment [136]. Delivering CRISPR tools plays a significant role in determining their safety and therapeutic effectiveness. Traditional GT using viruses has raised concerns about the potential for immune-related toxicity and insertional oncogenesis. However, AAV vectors continue to be a prominent option for CRISPR GT delivery because of their high delivery efficiency and are widely used for this purpose [137, 138].

### The most frequently utilized viral vectors for delivering CRISPR systems are AAVs

Due to their distinctive life cycle and interactions with host cells, recombinant AAVs have evolved into commercially viable gene therapies that serve as ideal genetic medicine instruments. One notable characteristic of AAVs is their capacity to modify the genome precisely. AAV, in contrast to all existing genome editing platforms, operates solely via the high-fidelity homologous recombination (HR) pathway, eliminating the need for exogenous nucleases to cleave genomic DNA beforehand. By combining these factors, an exceptionally accurate editing result is obtained, which maintains the integrity of the genome by preventing the introduction of viral sequences or indel mutations at the target site and eliminates the risk of off-target genotoxicity. It was discovered that AAVs derived from stem cells (AAVHSCs) mediated HR with high on-target accuracy and high efficiencies. In vivo, AAVHSC editing is effective in tissues and postmitotic cells. Moreover, AAV possesses the benefit of an intrinsic delivery mechanism. Therefore, this unique platform for altering the genome exhibits great potential in rectifying disease-associated mutations while preventing the accumulation of additional mutations. The distinct characteristics of direct AAV-mediated genome editing and their potential mechanisms of action will be the subject of this review [139, 140]. The advent of CRISPR genome editing technology facilitated the development of a multifaceted strategy for treating various diseases. Promising outcomes have been observed in many pre-clinical investigations and clinical trials. The combination of recombinant AAV and CRISPR technologies holds immense promise for developing therapeutic approach that irreversibly reverse genetic defects that cause disease. A desirable strategy, targeted insertion of a normal sequence to restore gene function, has extensive therapeutic potential irrespective of mutation type. Furthermore, despite the constrained packaging capacity of AAVs, the use of AAV for CRISPR delivery helps to minimize pathogenicity and naturally exhibits tissue specificity [141].

Gao et al. explored Cas13a, initially developed for virus detection, as a tool for gene interference in cancer treatment. They designed a Cas13a expression vector (DCUg) that used distinct promoters to regulate Cas13a and guide RNA (gRNA) expression. In experiments on human hepatoma cells (HepG2), DCUg effectively silenced reporter genes and oncogenes (TERT, EZH2, RelA), inducing apoptosis and inhibiting growth while sparing normal liver cells (HL7702). They also packaged this tool into AAV and demonstrated its effectiveness in inhibiting cancer cell growth in vitro and suppressing mouse tumor development. This study highlights the potential of CRISPR-Cas13a-based cancer GT delivered via AAV, showcasing its cancer cell-specific activity by selectively expressing Cas13a in tumor tissues. This approach holds promise for in vivo tumor GT, with potential therapeutic applications in the future [142].

Furthermore, the CRISPR/Cas system can potentially be a potent tool in safeguarding against carcinogenic viruses [143]. Patients with HCV who have baseline liver stiffness measurements show a correlation with the development of HCC [144]. Combining DNA engineering with RNAi expression technologies has shown promise in reducing the likelihood of HCC over an extended period. In a study conducted by Senis et al., The CRISPR/Cas9 system delivered employing AAV vectors, was utilized to precisely aim at the miR-122 locus and introduce an anti-HCV shmiRNA, a short hairpin RNA that is incorporated into miRNA into the genetic material. This Synergistic therapy, involving CRISPR/Cas and RNAi, demonstrated approximately 30% HDR-induced editing and a significant reduction of 10- to 100-fold in HCV viral replication in vitro [145].

## AAV challenges, disadvantages, and advantages in HCC

Some limitations of AAV-based GT vectors include their comparatively compact genetic material (4.7 kb), the infrequent chance of rAAV incorporation within the genetic framework of the host, and the potential existence of innate neutralizing antibodies targeting particular AAV serotypes in individuals [146]. One significant challenge in Liver-targeted GT is the widespread presence of neutralizing antibodies (NAbs), which can significantly reduce the effectiveness of treatments utilizing AAV when administered systemically. Even low concentrations of NAbs, including a ratio of 1:17 for AAV2 and 1:1 for AAV-Spark100 (a capsid artificially created from AAVrh74 origin), have been linked to decreased or even completely blocked therapeutic effectiveness. Consequently, a frequent exclusion criterion for individuals participating in clinical trials is the detection of NAbs against AAV capsids [147].

While rAAV vectors have demonstrated notable safety in liver-targeted GT, the potential for tumorigenesis due to inadvertent integration at unintended sites still exists. Although most AAV genomes remain episomal, infrequent integrations may take place, and with advancements in sequencing technology, they are becoming more accessible to detect. The initial indication that recombinant AAV might pose a risk of liver tumorigenesis originated from an investigation conducted by Donsante et al. [148], who observed an elevated prevalence of HCC in neonatal mice treated with AAV vectors. Upon investigation, they found that integrations, which increased in the tumors, primarily occurred in the Rian locus. The Rian locus is known to express several miRNA and small nucleolar RNAs [149]. Therefore, numerous independent investigations were conducted to assess the potential of AAV-mediated genotoxicity and tumorigenesis. The majority of these studies did not find elevated hazards in mature mice [150], or was not conclusively linked directly to the vector [151]. Research by Chandler et al. revealed that AAV often integrates into highly active liver genes [152]. Integration into the Rian locus was linked to HCC, and this connection depended on factors like dosage, the time of administration, and promoter potency. The prevailing opinion is that rAAV typically does not significantly heighten the cancer risk in adult mice. A decade-long investigation into AAV GT for canine hemophilia found clonal expansion among transduced hepatocytes after administering AAV8 and AAV9. Many integration events occurred in the vicinity of genes linked to cellular growth, subsequently resulting in elevated Factor VIII levels in a select group of animals. However, it's crucial to emphasize that tumors did not occur in any dogs due to these integrations [153, 154]. Notably, the AAV serotypes chosen for clinical studies are typically determined using data gathered from preclinical models, often in mice. Nevertheless, clinical data collected from AAV2, AAV5, and AAV8 usage demonstrates that AAV liver targeting, which can vary depending on the species [155], can show notable differences between preclinical models in mice or NHP and actual human patients [156]. It's crucial to recognize that the examination of the integration of the AAV genome into patients' livers treated with rAAV has revealed a safe profile over extended follow-up periods (over 12 years following vector delivery). There has been no indication of prolonged hepatic toxicity or the onset of HCC [157, 158]. Irrespective of the vector employed to deliver genetic material, it's imperative to monitor all patients to detect any possible cancerous integration thoroughly.. Additionally, targeting particular integration sites in safe genomic regions might offer a safer approach that ensures extended-term gene expression. Considering the complex and costly production process, it's essential to recognize that the liver-targeting capability of each AAV serotype can vary depending on the species [159].

On the contrary, rAAVs are highly appealing for therapeutic applications for several compelling reasons. First, there is a wide array of tested and dependable promoters available that can effectively drive the expression of the desired transgene. Second, rAAVs provoke minimal immune responses, and they can infect both quiescent and actively dividing cells, ensuring long-lasting genetic alterations. These qualities make them applicable to a broad range of disease models. Furthermore, AAV infections are not inherently linked to causing any disease, underscoring their safety record and suggesting that the virus is unlikely to induce significant side effects [160]. Additionally, owing to the natural propensity of several AAVs for the liver, HCC emerges as a compelling and robust target for AAV-based GT. Meumann and colleagues elucidated the inherent inclination of AAV2 vectors to infect HCC cells over nonmalignant liver cells, both in mouse models for studying HCC progression and in Studies involving precisely sectioned liver slices from human tissue (Table 2). With this natural tendency to target HCC, innovative treatment approaches can be devised, or current methods can be fortified to address the persistently bleak prognosis faced by most liver cancer patients [29].

**Table 2:** Benefits and drawbacks of employing AAV vectors in HCC gene therapy [161–163]

Advantages	Disadvantages
1. No immunogenicity (absence of viral coding sequences) 2. No inflammatory response from the host to capsid components 3. Effective penetration of DNA into the target cell 4. Extended DNA retention in the target cell	1. Necessitates conversion into double-stranded DNA, potentially causing a postponement in expression 2. Reduced integration frequency when Rep proteins are absent 3. Limited packaging capacity (up to a 4.7 kb insert) 4. Complicated production process and high cost

## Trials in the clinical setting for HCC therapy using AAV vectors

While there have been numerous clinical trials exploring AAV-based genetic therapy for various diseases, which have led to FDA approval of three AAV-assisted products (Zolgensma for spinal muscular atrophy, Luxturna for retinal dystrophy, and Hemgenix for hemophilia B), there are currently no active clinical trials focused on AAV-assisted genetic therapy aimed explicitly at treating HCC [164–166].

## The future for AAV vectors

With the promising preclinical results achieved using CRISPR/Cas systems, which enable precise genome editing at specific locations, the possibilities for AAVs in GT appear boundless [167]. Nevertheless, each approach has its drawbacks. AAV vectors have traditionally been perceived as relatively secure; however, recent evidence indicating that the potential for AAV vector genomes, when transporting CRISPR components, to incorporate within the host cell at double-strand break sites has prompted apprehensions about their efficacy and their safety over extended period [168]. Indeed, a decade-long follow-up investigation on six dogs that received GT vectors for F.VIII revealed the stable integration of vector genetic material into the host. This discovery has reignited concerns regarding the potential for oncogenic integration of AAV [23].

Conversely, extracellular vesicles (EVs) containing encapsulated AAVs offer a strategic means to bypass pre-existing resistance to viral vectors. The utilization of exosome-associated AAV (exo-AAV) vectors as a resilient hepatic gene delivery system is documented in a study. This approach reduces the therapeutic vector dose while safeguarding against preexisting humoral immunity to the capsid. The effectiveness of standard AAV8 or AAV5 and exo-AAV8 or exo-AAV5 vectors expressing human coagulation factor IX (hF.IX) in targeting the liver was assessed in vivo. A notable improvement in transduction efficacy was detected, and hemophilia B mice exposed to 4 × 1010 vector genomes per kilogram of exo-AAV8 vectors exhibited an astounding ∼1 log increase in hF. The observation of IX transgene expression resulted in enhanced correction of coagulation time. There was also a correlation between increased hepatic expression and a higher frequency of regulatory T cells in lymph nodes. Following this, the effectiveness of exo- and standard AAV8 vectors in eluding preexisting NAbs to the capsid was assessed in human sera and a passive immunization mouse model. The efficient transduction facilitated by exo-AAV8 gene delivery could potentially increase the proportion of eligible subjects for liver gene transfer, even in the presence of moderate NAb titers. Thus, exo-AAV vectors serve as a foundation for enhancing the effectiveness and safety of liver-directed gene transfer [169]. It is well established that the high prevalence of anti-AAV antibodies in humans, which precludes participation in GT trials, is a significant limitation of AAV-mediated gene transfer. A considerable percentage of individuals with natural humoral immunity to AAV, which varies by AAV serotype, possess low to moderate anti-capsid neutralizing titers. For instance, the investigators finding indicate that approximately 40% and 20% of healthy individuals, respectively, exhibit NAbs in a range of 1:1 to 1:3.16 for AAV2 and AAV8. While the titers as mentioned above are adequate to impede the targeting of the liver by conventional AAV vectors, our findings demonstrate that exo-AAV8 vectors remain capable of transducing the liver despite exposure to low to moderate concentrations of NAbs. An additional benefit of exo-AAV vectors is their ability to facilitate hepatic transduction in more potential patients [169–172]. While this delivery method offers several benefits, it has not yet been explored for transporting the CRISPR/Cas system [173].

Although AAV treatments have generally exhibited a commendable safety record in human applications, ongoing endeavors to assess both short-term and long-term safety profiles for AAV vectors remain imperative [174].

## Conclusion

As the incidence and mortality of HCC increase at an alarming rate around the globe, innovative, alternative therapies for HCC patients are urgently required. In the field of oncology, AAV vectors possess the capability to transduce an extensive array of cancer primary cells and cell lines. Moreover, they can transport therapeutic payloads that are exceptionally productive against cancer. These payloads may consist of DNA encoding smaller nucleic acids, anti-angiogenesis genes, suicide genes, and immunostimulatory genes. In addition, engineering AAV vectors to localize to primary and secondary tumors, as well as to tumor-initiating cells (sometimes referred to as "cancer stem cells"), which are resistant to conventional therapies and significantly contribute to the unfavorable prognosis and recurrence of many cancers after treatment, would be of great benefit. To sum up, exploring AAV vectors for HCC GT underscores their potential and challenges in this critical medical field. AAV vectors exhibit distinct advantages, including their non-immunogenic nature, efficient cell entry, long-term gene persistence, and tissue targeting capabilities. These vectors have shown safety in preclinical investigations and clinical trials, yet concerns persist regarding the risk of tumorigenesis and the influence of pre-existing immunity on therapeutic efficacy. Therefore, to successfully treat liver cancer, it is necessary to develop AAV or Ad vectors that target HCC with greater efficiency. Present approaches primarily concentrate on enhancing the therapeutic effectiveness of hybrid vectors, serotype substitution of distinct virus types, chemical modification of the virus capsid, and serotype substitution of various virus types to address HCC. The interplay of AAV vectors with cutting-edge technologies, notably CRISPR/Cas systems, promises a novel era of precision gene editing. In comparison, clinical trials using AAV-mediated GT have shown promise in diverse diseases, the absence of ongoing trials targeting HCC points to untapped potential in this domain. The future promises systemic administration, tissue-specific targeting, and programmable gene editing with minimal adverse effects. To fully actualize this capacity, it is imperative to continue rigorous evaluation of AAV vector safety and efficacy while integrating advances in delivery and genome editing technologies. Further research is warranted to evaluate the potential hazards of hepatocarcinogenesis induced by rAAV, considering its extensive application and promising potential in GT. In light of the shallow but potentially real risk that AAV vector integration may contribute to the development of HCC, individuals undergoing liver-directed AAV gene therapies should be thoroughly examined for past and future liver diseases.

In essence, the journey toward effective AAV-mediated GT for HCC is marked by triumphs and challenges, reflecting the intricate interplay of scientific discovery, translational application, and clinical prudence. The ongoing pursuit of excellence in this realm stands poised to unlock transformative therapies, underscoring the dynamic nature of GT's impact on the landscape of oncology and human health.

## Data Availability

Not applicable.
